# Disabling knee pain – another consequence of obesity: Results from a prospective cohort study

**DOI:** 10.1186/1471-2458-6-258

**Published:** 2006-10-19

**Authors:** Clare Jinks, Kelvin Jordan, Peter Croft

**Affiliations:** 1 Primary Care Musculoskeletal Research Centre, Primary Care Sciences, Keele University, Keele, Staffordshire, ST5 5BG, United Kingdom

## Abstract

**Background:**

Obesity is linked to knee osteoarthritis (OA) and knee pain. These are disabling problems that are more prevalent in older adults. No prospective study has estimated the impact of excess weight avoidance on the occurrence of knee pain in the general older population. The aim of this study was to investigate the influence of overweight and obesity on the onset and progression of knee pain and disability in older adults living in the community.

**Methods:**

A prospective cohort study of people aged 50 and over registered with three general practices in North Staffordshire, UK. 5784 people who had responded to a survey in March 2000 were mailed a follow-up questionnaire in March 2003. The main outcome measures were self-reported knee pain and severe knee pain and disability at 3 years measured by the Western Ontario and McMaster Universities Osteoarthritis index.

**Results:**

Adjusted response to follow-up was 75%. Among responders with no knee pain at baseline, obesity predicted onset of severe knee pain (relative risk 2.8; 95% CI 1.8, 4.5 compared to normal body mass index (BMI) category). Considering overweight and obese categories together, 19% of new cases of severe knee pain over a 3-year period could potentially be avoided by a one-category shift downwards in BMI; this includes almost half of the new cases that arose in the obese group.

**Conclusion:**

Obesity accounts for a substantial proportion of severe disabling knee pain. As knee pain is a common disabling condition in older adults living in the community, effective public health interventions about avoidance of excess weight could have a major impact on future lower limb disability in older adults.

## Background

Obesity is a worldwide health problem. The prevalence of obesity in adults in the United States has increased in the last 40 years from 13% to 31% [1]. Approximately two thirds of the population of England are overweight or obese and, in the last decade, obesity has increased by between 10 and 40% in Europe [2]. Globally, 300 million adults are obese [3].

Although much emphasis is placed on the link between obesity and life-threatening health problems like coronary heart disease and diabetes, another health consequence of obesity is knee osteoarthritis (OA) and disabling knee pain [4-9]. Although non-fatal, this musculoskeletal problem is a public health concern due to the scale of the problem and reduced quality of life it causes for increasing numbers of older adults. Ten percent of the world's population aged 60 or older have significant health problems attributed to OA [10] and the knee is the site most affected by joint pain in older adults [11,12]. The estimated 12-month prevalence of knee pain in the general older population is between 28 and 47% [13,14]. This common condition is, therefore, relevant to health care policy that aims to reduce the impact of disability on health and well-being, develop services that support independence or help avoid unnecessary admission to hospital.

The potential for weight loss to reduce the rate of symptomatic knee OA has been reported [15] but few studies [5,16] have attempted to estimate either the proportion of disabling knee pain in older people that might be attributed to pre-existing obesity, or the potential benefits of avoiding excess weight [7]. The aim of this study was to investigate prospectively the influence of obesity and overweight on (i) onset and (ii) progression of knee pain and related disability in adults aged 50 and over living in the community.

## Methods

All patients aged 50 and over registered at three general practices in North Staffordshire, U.K. were mailed a baseline questionnaire [6,14] in March 2000 and responders were mailed a 3-year follow-up survey. Both questionnaires included the Knee Pain Screening Tool (KNEST) which includes an enquiry as to whether the respondent has "had pain in or around the knee in the last year". A further question on the KNEST asks on how many days over the past 12 months the respondent had had this pain, and, using the answer, chronic knee pain was defined as more than three (not necessarily continuous) months of pain. A question on laterality asks whether the pain was in one (unilateral) or both (bilateral) knees. The screening tool was assessed for validity and reliability in a pilot study, the results of which have been previously reported [17].

Survey responders with knee pain also completed the Western Ontario McMaster Universities Osteoarthritis Index (WOMAC) [18] which asks about knee-related pain severity, stiffness and physical function over the previous 48 hours, and which has been validated for use in the general population [6]. As there was a high correlation between the pain and physical function scales scores (Pearson's correlation coefficient *r *= 0.84 at baseline) these two scales were combined and responders were defined as having severe knee pain or knee-related disability if they reported 'severe' or 'extreme' on at least one item on the pain scale or 'severe' or 'extreme' difficulty on at least one item on the physical function scale [6]. Participants who did not report any severe or extreme problem and answered at least 4 of the 5 pain items and 14 of the 17 physical function items were rated non-severe.

Self-reported height and weight at baseline were used to determine body mass index (BMI). BMI is calculated as weight in kilograms divided by the square of height in metres. Normal weight is defined as a BMI of 20–24.9, and in this study underweight was defined as a BMI under 20, a BMI of ≥ 25 and < 30 as overweight and 30 or over as obese [19]. As fewer than 5% of the baseline responders were defined as underweight and since BMI under 20 was unrelated to prevalence of knee pain at baseline, [6] individuals in the underweight category were combined with those in the normal weight category. This approach has been taken in previous studies of knee pain [4]. Test-retest reliability of self-reported BMI over two weeks in a pilot study on 59 subjects was substantial (based on Shrout's classification [20]; intraclass correlation coefficient 0.93, 95% confidence interval 0.88, 0.96).

Previous population studies [6,11,13] have highlighted the association of previous knee injury, pain elsewhere in the body, psychological distress and measures of social deprivation with knee pain and disability. Since these factors may also be linked with obesity, they were included as potential confounders of the link between obesity and knee pain and disability. Knee injury was based upon a question that asked whether responders had ever injured their knee badly enough to see a doctor about it. Widespread pain was captured from a body manikin on which responders shaded pain experienced in the last month. Widespread pain was defined as pain in the axial skeleton or lower back and in at least two areas of two contralateral limbs [21].

Psychological distress was measured by the Hospital Anxiety and Depression Scale [22]. This is a well validated psychological screening tool [23]. Responders scoring above the top tertile on the anxiety scale or depression scale were considered most anxious or most depressed.

Deprivation was measured using the Townsend deprivation score [24] derived from the 2001 U.K. Census. This is based on responder's postcode and combines variables on unemployment, overcrowding and car and home ownership. Responders scoring above the upper tertile of its distribution in the study population were classified as most deprived.

### Ethical Approval

Ethics approval for the project was gained from North Staffordshire Research Ethics Committee (LREC Project 862 for the baseline survey, LREC ref02/61 for the follow-up survey).

### Statistical analysis

The first set of analyses examined study aim (i), the influence of BMI category on onset of new knee pain. This was restricted to the group of participants who reported no knee pain at baseline. The incidence of any knee pain, and of severe knee pain, at three years (i.e. at follow-up) was calculated within each of the three BMI categories (normal, overweight and obese). The risks of any incident knee pain, and of incident severe knee pain, were calculated for the overweight and obese groups relative to the normal group.

The relative risks were calculated first unadjusted, and then adjusted for age, gender, deprivation, depression, previous knee injury and widespread pain, all measured at baseline. These variables were all related to presence of knee pain at baseline. The adjusted relative risks were obtained using Cox regression models with a constant time variable [25].

An Attributable Fraction (AF) and Population Attributable Fraction (PAF) approach was used. The AF was first used to determine the fraction of incident knee pain in people exposed to obesity or overweight which would not have occurred in the absence of the exposure [26]. The PAF was used to calculate the proportion of incident cases of knee pain in the population that could be avoided if the exposures (in this case overweight and obesity) were removed.

The AF was calculated for a shift in the overweight population from overweight to normal weight, and of the obese study population from obese to overweight. The AF for overweight was calculated first, as r−1r
 MathType@MTEF@5@5@+=feaafiart1ev1aaatCvAUfKttLearuWrP9MDH5MBPbIqV92AaeXatLxBI9gBaebbnrfifHhDYfgasaacH8akY=wiFfYdH8Gipec8Eeeu0xXdbba9frFj0=OqFfea0dXdd9vqai=hGuQ8kuc9pgc9s8qqaq=dirpe0xb9q8qiLsFr0=vr0=vr0dc8meaabaqaciaacaGaaeqabaqabeGadaaakeaadaWcaaqaaiabdkhaYjabgkHiTiabigdaXaqaaiabdkhaYbaaaaa@3173@ where *r *is the risk of developing knee pain in those exposed (i.e. overweight) relative to people not exposed (i.e. normal weight). The number of cases of incident knee pain in the overweight group which might have been avoided was then calculated by multiplying the AF by the number of incident cases in the exposed. This calculation was repeated with obesity defining the exposed group and the overweight group as the unexposed. The total number of incident cases in the study population which could have been avoided was determined by adding the two results and this was expressed as a percentage of all incident cases in the study population (the Population Attributable Fraction (PAF)). The same approach was then adopted for calculating AF and PAF for severe knee pain.

The second set of analyses examined study aim (ii), the influence of BMI category on progression from non-severe knee pain at baseline to severe pain 3 years later. The relative risks (unadjusted and adjusted) of progression to severe knee pain were calculated for the overweight and obese groups compared to the normal BMI group. In the adjusted analysis, chronicity and laterality of knee pain at baseline were added to the potential confounding variables. The attributable fraction approach described above was then repeated for progression to severe knee pain.

Finally, results from this second set of analyses were combined with the results for incident severe knee pain amongst those with no knee pain at baseline in order to calculate a PAF for all new severe knee pain in the study population.

Analysis was performed using SPSS Inc 12.0 for Windows (SPSS inc, Chicago, Illinois).

## Results

At baseline, 8995 people were sent a questionnaire, to which 6792 responded. After taking into account deaths and departures (e.g. people moving away from practices), the adjusted baseline response was 77%. Response was higher in females (79%) than males (75%, chi-square test, *p *< 0.001) and responders were slightly older than non-responders (mean difference 1.5 yrs, 95% CI 1.0, 2.0). Prior to follow-up, 860 baseline responders had died or moved away and another 148 were excluded as they were now involved in another local study. Overall, 5784 people were sent a follow-up questionnaire of which 4317 (75%) responded.

3769 (87%) of these responders answered the question on knee pain at both baseline and follow-up and were able to have a baseline BMI calculated. Compared to the other 2015 who were sent a follow-up questionnaire, these 3769 responders were slightly younger (mean difference 2.1 yrs; 95% CI 1.6, 2.6), and included a slightly higher proportion of males (44% v. 42%, *p *= 0.052). However, they were no different in terms of prevalence of knee pain at baseline (*p *= 0.71) or percentage rated overweight or obese (*p *= 0.24). Baseline characteristics of these 3769 people are given in table 1.

### Onset of new knee pain among responders with no knee pain at baseline

The incidence of any knee pain at the three-year follow-up was 24% among responders with no knee pain at baseline, ranging from 23% of the normal weight category to 31% of the obese group (table 2). Table 2 shows a modest association of obesity with subsequent onset of knee pain (unadjusted RR 1.39; 95% CI 1.11, 1.74 compared with normal BMI category), but no association of overweight with any new knee pain. Thirty of the 483 cases of new knee pain (or 6.2% of all incident cases over a three year period) were attributable to overweight or obesity at baseline. After adjusting the relative risks for age, gender, depression, previous knee injury and widespread pain, the association with obesity weakens and becomes statistically non significant (RR 1.26, 95% CI 0.95, 1.67, compared with the normal weight category), and number of cases attributable falls to 25 (5.2% of all incident cases).

Among responders with no knee pain at baseline, 7% developed severe knee pain at three years. Obesity was a strong predictor of onset of severe knee pain in this group, overweight less so (table 3) (adjusted RR for obesity compared to the normal BMI category is 2.79, 95% CI 1.75, 4.47; adjusted RR for overweight compared to the normal category is 1.53, 95% CI 1.03, 2.26). Thirty-five cases of severe knee pain over a three-year period could have been prevented by avoiding excess weight (although some might still have developed non-severe knee pain). This is equivalent to 25% of all incident cases among responders with no knee pain at baseline.

### Progression of knee pain among responders with non-severe knee pain at baseline

Among participants with non-severe knee pain at baseline, 19% had developed severe knee pain at three years. Obesity was a predictor of severe knee pain at three years in this group (table 4 – adjusted RR 1.77; 95% CI 1.16, 2.69 compared to the normal BMI category). Being overweight rather than normal weight was not associated with future severe knee pain in this group (RR 1.07; 95% CI 0.74, 1.56). An estimated 18 cases of severe knee pain at follow-up among participants who had reported non-severe knee pain at baseline could be attributed to obesity. This is 11% of all incident cases which occurred in the non-severe group.

### Population potential for reducing disabling knee pain in older adults

The combined results of tables 3 and 4 are shown in table 5. Overall incidence of severe knee pain at the third year of follow-up (new pain or progression) was 11%. An estimated 19% of all incident cases of severe knee pain over a 3 year period (*n *= 57 in this sample) could be attributed to obesity or being overweight. This includes 33 (43%) of the 77 cases in the obese group. The main effect of being overweight was in those with no, rather than non-severe, knee pain at baseline.

## Discussion

Amongst older adults without knee pain, those who were obese were nearly three times more likely than those of normal weight to develop severe knee pain in a subsequent three-year period. Including progression from non-severe pain, almost one-fifth of all new cases of severe knee pain in people aged 50 years and over in a three-year period could be avoided if excess weight was prevented.

The implications of our findings for public health, clinicians and researchers are clear as they illustrate how the population burden of knee pain could diminish if the proportion of overweight and obese people (i.e. those with a BMI ≥ 25) in the total population of older people were to decline. Being obese or overweight were predictors of onset of severe knee pain at three years in responders who were free of knee pain at baseline. Avoidance of excess weight, in this group in particular, is likely, therefore, to assist with primary prevention of knee pain in the older population. As knee pain is a common and disabling condition in older adults living in the community, the potential for health gain in the population is large.

Our findings add to previous longitudinal research on the link between obesity and knee OA [8]. Gelber and colleagues studied male medical students (median follow-up 36 years) and found that a greater BMI in men aged between 20 to 29 was associated with an increased risk of subsequent knee OA [8]. Our population-based findings reinforce the need for early primary prevention. In our study, obesity (defined as BMI >30) was also a strong predictor of progression of non-severe knee pain to severe knee pain at three years. Public health interventions targeted at avoiding excess weight in those with non-severe knee pain are likely to assist with secondary prevention.

Our study was not an intervention study. Our analyses have not taken into account the effect of any weight change. It cannot be concluded from our study that weight loss, rather than avoiding excess weight in the first place, would reduce the amount of severe and disabling knee pain in older people. However, weight loss is recommended as treatment for knee osteoarthritis [27]. Given that osteoarthritis is a chronic but generally non-fatal disease, people with established severe knee pain and disability might usefully use weight reduction to manage and alleviate these symptoms. So it is likely that our estimates of the impact of avoiding excess weight, which are based on the incidence of new pain or the onset of severe or disabling knee pain in the general population, are underestimates of the total impact which interventions aimed at both excess weight avoidance and weight reduction in the population might have on incident and prevalent problems combined. Our results suggest, however, that the main advantage in population terms is in preventing obesity; the excess risk is small in the overweight group, and so, despite the fact that the overweight category contains more people overall, the number of potentially preventable cases in this group is small compared to those generated by the obese group.

In our study, responders in the obese category would have to reduce their weight by a median of 13.5 pounds (6.1 kg) to move into the overweight category. Those overweight would have to reduce their weight by a median of 12.4 pounds (5.6 kg) to move into the normal weight category. In a study exploring the effects of actual weight loss, a loss of approximately 5.1 kg decreased the odds of developing symptomatic knee OA by 50% [15].

Identifying and implementing effective interventions for avoiding weight gain or achieving weight loss in the older population remains a challenge. There is evidence that long-term weight loss needs to be supplemented with exercise [28]. Barriers to physical activities that are experienced by older adults (for example, fear of pain, misconceptions about benefits of exercise, environmental factors) [29] must also be addressed. There is evidence of beneficial effects of exercise per se in knee OA patients [30] so providing information about the benefits of both weight loss and exercise to the community is a realistic start. Mehrortra and colleagues studied the prevalence of professional advice to lose weight among obese adults with arthritis who had a recent physician visit [31]. Less than half of obese arthritis sufferers recalled receiving advice on weight loss at their last visit. Providing information is a short-term strategy but in the long-term a wider public health approach will be required.

Approximately 98% of the British population is registered with a GP [32] and the register provides a convenient sampling frame of a local population. The prevalence of knee pain at baseline was 46.8% [14]. To take account of non-response bias at baseline we standardised this to the age and gender distribution of the entire older population of the participating general practice registers. The population estimate of prevalence was unchanged by this. As responders were slightly older than non-responders at baseline, but slightly younger at follow-up, the age of our sample was similar at time of follow-up to the original study population.

Based on mid-2000 population estimates, [33] the demographic profile of the study sample is similar to that of North Staffordshire as a whole (the region in which the 3 GP practices were based) and of England and Wales. In our study, 56% of baseline responders were female, compared with 54% of the over 50 population in North Staffordshire and England and Wales. The proportion of people aged over 75 years in the baseline survey sample was 21%, compared to 22% in North Staffordshire and 23% in England and Wales. The study sample was slightly different to the UK as a whole, however, in terms of ethnicity as 99% of responders were white UK/European origin, a figure that does reflect the make up of the North Staffordshire population. The potential for prevention of knee pain may be different in populations with a different ethnic mixture. However, we estimate that 19% of new cases of severe knee pain could be avoided by a shift downwards in BMI category and this is similar to that derived in a US study by Leveille et al [16] who reported 18% of arthritis cases were attributable to obesity.

This is a population-based study and our aim was to report the impact of excess weight avoidance on knee pain in the general population, regardless of consultation. This approach required the use of self-reported knee pain data instead of, for example, radiographic evidence of changes within the knee joint. The Knee Pain Screening Tool has been previously reported as valid and reliable [17]. However, individuals with musculoskeletal pain often experience episodes of pain separated by pain free periods. The two surveys used in this study may have failed to capture some such recurrently painful episodes, thereby introducing some underestimation of episodic knee pain.

We also used self-reported data to calculate BMI and this may underestimate true population BMI [34]. However, the mean BMI score in our population was is in line with national trends [35]. The test-retest reliability of our BMI data was very good. Any misclassification would mean that our risk ratios are underestimates of the true associations between knee pain and obesity and overweight.

Another limitation is that there may be confounding factors for which our analysis has been unable to account. Such influences might include, for example, the use of medications, occupation, physical activities and other health problems like cardiovascular disease.

## Conclusion

The implication from this prospective cohort study is that there is a clear potential to reduce the burden of disabling knee pain in the population if the excess associated, in particular, with obesity was removed by a small shift in the weight of the older population. Since musculoskeletal conditions contribute more to poor quality of life than other chronic conditions [36] reducing this burden will have substantial implications for the health of older people. The challenge now is one of testing and implementing interventions to achieve this.

## Competing interests

All authors are independent of the funders. The authors declare that they have no competing interests.

## Authors' contributions

All authors contributed to this manuscript. CJ conceived the design of the study, coordinated the study and participated in revision of the manuscript and interpretation of data. KJ undertook analysis and participated in drafting the manuscript and in interpreting the data. PC participated in drafting the manuscript and in interpreting the data. All authors read and approved the final manuscript.

## Pre-publication history

The pre-publication history for this paper can be accessed here:



## Figures and Tables

**Table 1:** Baseline characteristics of sample (*n *= 3769)

	*n *(%)
Male Female	1674 (44) 2095 (56)
Age group	
50–64	2031 (54)
65–74	1194 (32)
75+	544 (14)
BMI^a^	
normal (20–24.9)	1577 (42)
overweight (≥25 and < 30)	1584 (42)
obese (≥30)	608 (16)
Anxious	1015 (27)
Depressed	818 (22)
Reside in deprived area	1189 (32)
Widespread pain	353 (9)
Previous knee injury	969 (26)
Any knee pain^b^	1773 (47)
Severe knee pain^c^	770 (21)
Chronic knee pain^d^	928 (25)
Bilateral knee pain^e^	876 (23)

^a ^BMI = body mass index (normal weight category includes individuals who were underweight).

^b^Based on KNEST knee pain screening question: Have you had pain in the last year in or around the knee?

^c^Based on responders to ^b ^(above) who also completed the WOMAC.

^d^Defined as more than three (not necessarily continuous) months of pain.

^e^Defined as pain in both knees

**Table 2:** Onset of knee pain at three years among people aged 50 and over with no knee pain at baseline by body mass index (BMI) category.

BMI category at baseline		Knee Pain at 3 years	Unadjusted analysis			Adjusted analysis^a^		
	*n*	*n *(%)	Relative Risk (RR) (95% Confidence Interval (CI))	RR^b ^(95% CI)	Potential cases avoided^c ^(% of all incident cases)	RR (95% CI)	RR^b ^(95% CI)	Potential cases avoided^c ^(% of all incident cases)
Normal^d^	960	216 (22.5)	1.00	-	-	1.00	-	-
Overweight	809	196 (24.2)	1.08 (0.91, 1.28)	1.00	14 (2.9)	1.08 (0.89, 1.32)	1.00	15 (3.1)
Obese	227	71 (31.3)	1.39 (1.11, 1.74)	1.29 (1.03, 1.62)	16 (3.3)	1.26 (0.95, 1.67)	1.17 (0.88, 1.56)	10 (2.1)
Total	1996	483 (24.2)			30 (6.2)			25 (5.2)

^a ^adjusted for age, gender, deprivation, anxiety, depression, previous knee injury, widespread pain measured at baseline

^b ^obesity category compared to overweight

^c ^number of cases which could have been avoided by reducing overweight people to normal and obese people to overweight

^d ^Normal weight category includes individuals who were underweight.

**Table 3:** Onset of severe knee pain at three years in people aged 50 and over with no knee pain at baseline by body mass index (BMI) category.

BMI category at baseline		Severe knee pain at 3 years	Unadjusted analysis			Adjusted analysis^a^		
	*n*	*n *(%)	Relative Risk (RR) (95% Confidence Interval (CI))	RR^b ^(95% CI)	Potential cases avoided^c ^(% of all incident cases)	RR (95% CI)	RR^b ^(95% CI)	Potential cases avoided^c ^(% of all incident cases)
Normal^d^	939	50 (5.3)	1.00	-	-	1.00	-	-
Overweight	785	56 (7.1)	1.34 (0.93, 1.94)	1.00	14 (10.1)	1.53 (1.03, 2.26)	1.00	20 (14.4)
Obese	223	33 (14.8)	2.78 (1.84, 4.21)	2.07 (1.39, 3.11)	17 (12.2)	2.79 (1.75, 4.47)	1.85 (1.17, 2.92)	15 (10.8)
Total	1947^e^	139 (7.1)			31 (22.3)			35 (25.2)

^a ^adjusted for age, gender, deprivation, anxiety, depression, previous knee injury, widespread pain measured at baseline

^b ^obesity category compared to overweight

^c ^number of cases which could have been avoided by reducing overweight people to normal and obese people to overweight

^d ^Normal weight category includes individuals who were underweight

^e ^Total number is smaller than in Table 2, because 49 people had incomplete WOMAC data and severity scores could not be calculated.

**Table 4:** Onset of severe knee pain at three years in people aged 50 and over with non-severe knee pain at baseline by body mass index (BMI) category.

BMI category at baseline		Severe knee pain at 3 years	Unadjusted analysis			Adjusted analysis^a^		
	*n*	*n *(%)	RR (95% CI)	RR^b ^(95% CI)	Potential cases avoided^c ^(% of all incident cases)	RR (95% CI)	RR^b ^(95% CI)	Potential cases avoided^c ^(% of all incident cases)
Normal^d^	356	56 (15.7)	1.00	-	-	1.00	-	-
Overweight	383	67 (17.5)	1.11 (0.80, 1.54)	1.00	7 (4.2)	1.07 (0.74, 1.56)	1.00	4 (2.4)
Obese	148	44 (29.7)	1.89 (1.34, 2.67)	1.70 (1.22, 2.36)	18 (10.8)	1.77 (1.16, 2.69)	1.66 (1.10, 2.52)	18 (10.8)
Total	887	167 (18.8)			25 (15.0)			22 (13.2)

^a ^adjusted for age, gender, deprivation, anxiety, depression, previous knee injury, widespread pain, chronicity of knee pain (3 months or more) at baseline and laterality of knee pain (1 knee or both) at baseline

^b ^obesity category compared to overweight

^c ^number of cases which could have been avoided by reducing overweight people to normal and obese people to overweight

^d ^Normal weight category includes individuals who were underweight.

**Table 5:** Onset of severe knee pain at three years in adults aged 50 years and over with no knee pain or non-severe knee pain at baseline by body mass index (BMI) category.

BMI category at baseline		Severe knee pain at 3 years	Unadjusted analysis	Adjusted analysis^a^
	*n*	*n *(%)	Potential cases avoided^b ^(% of all incident cases)	Potential cases avoided^b ^(% of all incident cases)
Normal^c^	1295	106 (8.2)	-	-
Overweight	1168	123 (10.5)	21 (6.9)	24 (7.8)
Obese	371	77 (20.8)	35 (11.4)	33 (10.8)
Total	2834	306 (10.8)	56 (18.3)	57 (18.6)

^a ^adjusted for age, gender, deprivation, anxiety, depression, previous knee injury, widespread pain, chronicity of knee pain (3 months or more) at baseline and laterality of knee pain (1 knee or both) at baseline

^b ^number of cases which could have been avoided by reducing overweight people to normal and obese people to overweight

^c ^Normal weight category includes individuals who were underweight.
